# Study of ALDH from *Thermus thermophilus*—Expression, Purification and Characterisation of the Non-Substrate Specific, Thermophilic Enzyme Displaying Both Dehydrogenase and Esterase Activity

**DOI:** 10.3390/cells10123535

**Published:** 2021-12-14

**Authors:** Kim Shortall, Edel Durack, Edmond Magner, Tewfik Soulimane

**Affiliations:** 1 Department of Chemical Sciences, Bernal Institute, University of Limerick, V94 T9PX Limerick, Ireland; kimleah.shortall@ul.ie (K.S.); edmond.magner@ul.ie (E.M.); 2 Department of Biological Sciences, University of Limerick, V94 T9PX Limerick, Ireland; edel.durack@ul.ie

**Keywords:** aldehyde dehydrogenase, heat treatment purification, substrate specificity, esterase activity, biochemical characterisation, thermophilic enzyme

## Abstract

Aldehyde dehydrogenases (ALDH), found in all kingdoms of life, form a superfamily of enzymes that primarily catalyse the oxidation of aldehydes to form carboxylic acid products, while utilising the cofactor NAD(P)^+^. Some superfamily members can also act as esterases using *p*-nitrophenyl esters as substrates. The ALDH_Tt_ from *Thermus thermophilus* was recombinantly expressed in *E. coli* and purified to obtain high yields (approximately 15–20 mg/L) and purity utilising an efficient heat treatment step coupled with IMAC and gel filtration chromatography. The use of the heat treatment step proved critical, in its absence decreased yield of 40% was observed. Characterisation of the thermophilic ALDH_Tt_ led to optimum enzymatic working conditions of 50 °C, and a pH of 8. ALDH_Tt_ possesses dual enzymatic activity, with the ability to act as a dehydrogenase and an esterase. ALDH_Tt_ possesses broad substrate specificity, displaying activity for a range of aldehydes, most notably hexanal and the synthetic dialdehyde, terephthalaldehyde. Interestingly, *para*-substituted benzaldehydes could be processed efficiently, but *ortho*-substitution resulted in no catalytic activity. Similarly, ALDH_Tt_ displayed activity for two different esterase substrates, *p*-nitrophenyl acetate and *p*-nitrophenyl butyrate, but with activities of 22.9% and 8.9%, respectively, compared to the activity towards hexanal.

## 1. Introduction

Found in all kingdoms of life, aldehyde dehydrogenases (ALDH) (EC;1.2.1.3) constitute a large family of NAD(P)^+^-dependent enzymes with a molecular mass of ca. 50–60 kDa, and are composed of 450–500 amino acids. ALDHs exist as dimers [1], tetramers [2,3,4] and hexamers [5]; however, the latter are less prevalent, with only two resolved structures available to date [6,7,8]. Their structures consist of three conserved domains, the catalytic domain, the cofactor binding domain, and the oligomerisation domain [1], which work together to catalyse the conversion of an aldehyde substrate using NAD(P)^+^ cofactor to form the corresponding carboxylic acid and NAD(P)H. Additionally, some ALDHs can act as esterases [9,10] or can reduce nitrate [11], indicating that this family of enzymes possess broad catalytic properties. There is evidence that dehydrogenase and esterase activity of ALDH occurs within the same active site, utilising the same catalytic residues [12,13]. The dehydrogenase activity of ALDH occurs in five distinct steps [13] (demonstrated with human numbering): (1) activation of the catalytic thiol, Cys302, using a water molecule for water-mediated deprotonation by Glu268; (2) consequential nucleophilic attack on the electrophilic aldehyde by the thiol group of the catalytic cysteine; (3) formation of a tetrahedral thiohemiacetal intermediate, via deacylation, coupled with concomitant hydride transfer to the pyridine ring of NAD(P)^+^; (4) hydrolysis of the resulting thioester; and (5) dissociation of the reduced cofactor with concomitant regeneration of the enzyme via NAD(P)^+^ binding [13]. Esterase activity also includes five steps utilising the same catalytic residues: (1) Cys302 is activated by hydrogen abstraction from Glu268; (2) thiolate ion attacks the *p*-nitrophenyl ester substrate creating an oxyanion intermediate; (3) the intermediate rearranges and the nitrophenol group leaves; (4) Glu268 abstracts hydrogen from an ordered water molecule making it a nucleophile and attacks the carbonyl in the thioacyl enzyme complex; and (5) formation of the tetrahedral intermediate and release of the carboxylic acid and free enzyme [13].

**Scheme 1:**
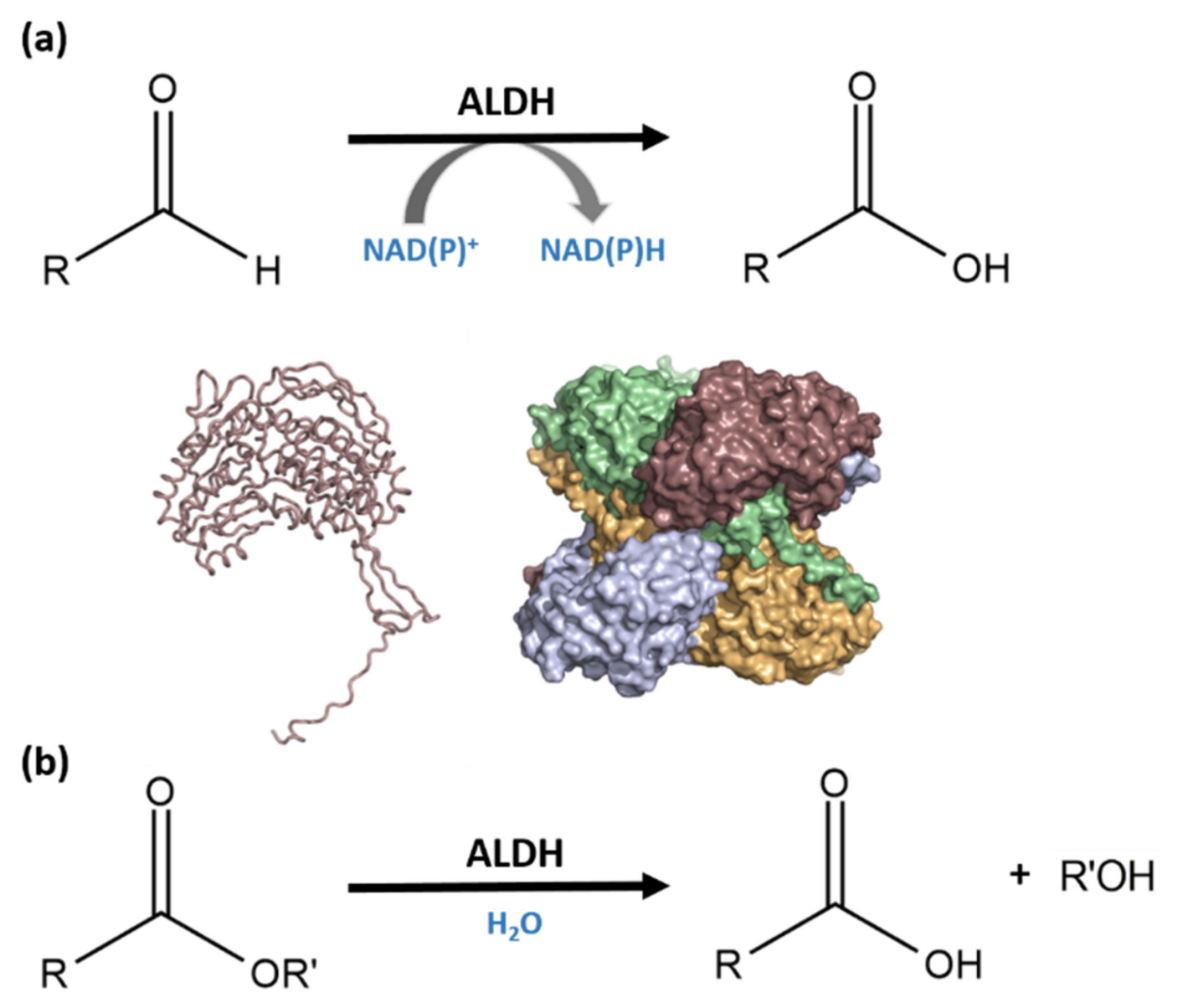
ALDH reaction mechanism highlighting ALDH_Tt_ monomeric and tetrameric structure (PDB ID: 6FJX) (**a**) dehydrogenase activity using cofactor, (**b**) esterase activity [14].

ALDH enzymes catalyse the conversion of an aldehyde to carboxylic acid with the requirement of cofactor, whereas hydrolysis of an ester can be achieved by ALDH without NAD(P)^+^ (Scheme 1). Previous studies highlight that the addition of a cofactor can enhance or inhibit esterase activity without being directly involved in the catalytic process [9,14,15]. Human ALDH1 and 2 isozyme’s esterase activity is accelerated with addition of cofactor, while the activity of ALDH3 is inhibited [15].

ALDHs are often not substrate specific, and can oxidise a wide range of aliphatic and aromatic, endogenous and exogenous aldehydes [16,17,18], which might be related to their broad spectrum of associated biological functions [5]. The primary function of ALDHs is their role in detoxification mechanisms [19,20,21], while other functions include biosynthesis [22,23] and non-enzymatic roles, such as antioxidant [24,25], structural [26], and regulatory function [27,28]. 

While ALDHs have been well-characterised in humans, emerging research places a primary focus on their prokaryotic counterpart’s structure and function, which has been less explored until recently [29,30,31]. The scope of prokaryotic ALDH function spans much wider than eukaryotes [5,19,31,32]. For example, the *E. coli* enzymes phenylacetaldehyde dehydrogenase and lactaldehyde dehydrogenase were characterised after the ALDH1, 2, and 3 from humans [1,2,33], and are involved in the specific metabolic pathways of phenylalanine and fucose respectively. Other examples include a long-chain alkane degrading ALDH from the thermophilic *Geobacillus thermoleovorans* (ALDH_Gtv_) and betaine biosynthesis in *E. coli* [34,35]. Recently, characterisation of prokaryotic ALDHs has led to understanding of niche features and characteristics within the superfamily, such as extensions consisting of non-functional domains [29]. Interestingly, in the last decade, the presence of retinoic acid within prokaryotes was discovered [36,37], highlighting the possibility of retinoid signalling pathways dependent on ALDH and cytochrome oxidases for the conversion of retinaldehyde to retinoic acid. These mechanisms, until recently, were thought to be only of animal origin, but emerging research suggests the contrary [36]. 

Many ALDHs possess broad substrate specificity, and, in some cases, the true biological substrate(s) has not been identified. Human ALDH1 family members are primarily characterised as retinaldehyde oxidising isozymes. A recent study demonstrated that ALDH1A members could process medium chain aliphatic compounds as well as retinaldehyde [22]. The ALDH from *Geotrichum candidum* (ALDH_Gc_) displayed catalytic activity with an extensive range of aldehyde substrates [16]. ALDH_Gc_ demonstrated conserved or increased specific activity for a spectrum of *para*-substituted benzaldehydes, short to medium chain aliphatics (acetaldehyde to heptanal), and dialdehydes, including terephthalaldehyde with respect to benzaldehyde. Thermophilic ALDHs have also proven to have broad substrate specificity. ALDH_Gtv_ can process medium to long chain aldehydes, while ALDH from *Geobacillus thermodenitrificans* (ALDH_Gtd_) and *Pseudomonas putida* (ALDH_Pp_) exhibited activity for 15 and 21 different aromatic and aliphatic substrates respectively, working optimally at 60 °C [38,39]. The active site of ALDH_Pp_ is highly hydrophobic, linked to the enzyme preferring non-polar substrates and converting them more efficiently, e.g., acetaldehyde is the most inefficiently processed. ALDH_Pp_ could even process the pyrene ring derived pyrene-1-carboxaldehyde. 

ALDH enzymes have the potential to act as a biocatalytic route for carboxylic acid synthesis [40]. Traditional methods for the oxidation of aldehydes to carboxylic acids is no longer sustainable due to the use of stoichiometric amounts of transition metal oxidants, salts or Ag_2_O in combination with sodium cyanide, besides complicated reaction protocols. Biocatalytic methods employing enzymes are attractive due to the use of mild reaction conditions coupled with the regio- and chemo-selectivity. Investigation of ALDHs in biocatalysis has only been marginally investigated, but their exquisite chemoselectivity and broad substrate scope makes them attractive candidates [40,41,42]. Additionally, there is a considerable demand for more stable and better performing catalysts, to which an efficient solution is the use of thermophilic enzymes [43]. 

Previously, the structure of ALDH_Tt_ was resolved revealing a tetrameric organisation (Scheme 1) with a distinct C-terminal tail which plays a role in oligomerisation, active site regulation, and thermostability [30]. The tail wraps the opposing monomer in a diagonal fashion, and as it does so it is dragged across the substrate entry channel with possible occlusion of the active site. Even though the structure of this enzyme has been determined, its biochemical characterisation is important to understand and link the structure and function. Herewith, we describe the recombinant expression, purification, and biochemical characterisation of the ALDH_Tt_ from *T. thermophilus*. The aim is to efficiently purify the thermophilic ALDH_Tt_ to allow for the investigation of its catalytic ability in terms of substrate specificity and reaction mechanisms. Both dehydrogenase and esterase activity are analysed with a range of possible substrates for enzymatic characterisation of the ALDH_Tt_ providing insight into its possible biological roles or use in biocatalysis.

## 2. Materials and Methods

### 2.1. Protein Sequence Alignment and BLAST Analysis

Protein sequences were retrieved from the NCBI database (National Center for Biotechnology Research) (https://www.ncbi.nlm.nih.gov/ accessed on 1 November 2021) and aligned in multiple sequence format using the Clustal Omega multiple sequence alignment tool (https://www.ebi.ac.uk/Tools/msa/clustalo/ accessed on 5 November 2021). The protein sequence alignments were visualised using GeneDoc software. The basic local alignment search tool (BLAST) was carried out for ALDH_Tt_ utilising the NCBI’s protein BLAST search (https://blast.ncbi.nlm.nih.gov/Blast.cgi accessed on 9 December 2021). Sequences for alignment were obtained from NCBI for ALDH_Tt_ (6FJX), human ALDH1A3 (5FHZ) and succinate semialdehyde dehydrogenase (SS-ALDH) from *E. coli* (3JZ4).

### 2.2. Native ALDH_Tt_ Purification with caa_3_-Oxidase 

Crystalline native ALDH_Tt_ was first identified as an impurity, during the crystallisation of the *caa_3_*-cytochrome c oxidase [30,44]. ALDH_Tt_ was purified from the *caa_3_*-oxidase through cation exchange chromatography and ammonium sulfate precipitation. 

### 2.3. ALDH_Tt_ Recombinant Expression and Purification 

The construct DNA pET-22b(+)-ALDH_Tt_ [30] was transformed via heat shock at 42 °C into *E. coli* BL21(DE3) competent cells (Invitrogen). Expression of ALDH_Tt_ was performed in ZYP-5052 auto induction media, supplemented with ampicillin (50 μg/mL), and inoculated with 1% (v/v) of overnight culture of transformed cells in LB broth grown at 37 °C. Expression was carried out for 48 h at 25 °C, 200 rpm, with subsequent cell collection at 6000× *g*, 4 °C for 15 min. The cell pellet was resuspended in lysis buffer containing 20 mM Tris-HCl pH 7.5, 5 mM β-mercaptoethanol, 10 mM imidazole, and 500 mM NaCl, with 5 mL added per g of cells. The lysis buffer was supplemented with 0.25 mg/mL lysozyme, 20 μg/mL DNase I, and 200 mM MgCl_2_, and frozen at −80 °C overnight. Following overnight freezing, the cells were thawed, sonicated, heat treated at 65 °C for 15 min, and collected by centrifugation at 17,000 rpm, 4 °C for 30 min. The soluble fraction was filtered through a 0.45 μm nylon filter and loaded onto an XK 16/20 column containing chelating Sepharose fast flow activated with 0.2 M NiSO_4_, and pre-equilibrated with 20 mM Tris-HCl pH 7.5, 5 mM β-mercaptoethanol, 10 mM imidazole, and 200 mM NaCl for affinity chromatography. Bound proteins were eluted using a step gradient of imidazole at concentrations of 50, 100, 200, and 500 mM in 20 mM Tris-HCl, 5 mM β-mercaptoethanol and 150 mM NaCl, pH 7.5. Fractions containing the ALDH_Tt_ were dialysed overnight at 4 °C against 50 mM Tris-HCl, 5 mM β-mercaptoethanol and 250 mM NaCl, pH 7.5, and concentrated using Amicon Ultra-15 centrifugal filters, 50 kDa MWCO (Merck Millipore). Approximately 1 mL of concentrated protein was loaded onto a HiLoad 16/60 Superdex 200 pg column, pre-equilibrated with 50 mM Tris-HCl pH 7.5, 5 mM β-mercaptoethanol and 150 mM NaCl. The eluted fraction was concentrated to a desired concentration (25–30 mg/mL), snap-frozen in liquid nitrogen, and stored at −80 °C until further use. 

### 2.4. SDS-PAGE and Western Blot

Protein samples were run on a 12% SDS-PAGE gel, stained using Instant Blue (Sigma) and destained in deionised water overnight. For Western blot, proteins were transferred from an unstained gel to nitrocellulose membrane at 80 mAmp for 90 min, followed by subsequent blocking and washing steps. The ALDH_Tt_ was coupled with an anti-6xhis-HRP antibody, followed by colorimetric detection utilising the substrate 5-Tetramethylbenzidine (TMB). 

### 2.5. MALDI-TOF Mass Spectrometry 

The molecular mass of the ALDH_Tt_ was confirmed by matrix-assisted laser desorption ionisation-time of flight (MALDI-TOF) mass spectrometry using α-cyano-4-hydroxycinnamic acid (HCCA) as the matrix. The ALDH_Tt_ (31.12 mg/mL) was diluted 1 in 30 in water to minimise the presence of salts before analysis. A 1 µL aliquot of protein solution was added to 1 µL of the HCCA matrix, with 1 µl of sample then added to the steel sample plate. The sample plate was inserted into the MALDI-TOF Ultraflex mass spectrometer (Bruker) and run in linear cationic 5–60 kDa mode, using 2500 shots. The theoretical molecular mass was determined using Expasy online molecular weight calculation tools (https://web.expasy.org accessed on 1 November 2021) and amino acid sequencing of ALDH_Tt_ (6FJX) from NCBI.

### 2.6. Activity Assessment–Temperature and pH

The catalytic activity of ALDH_Tt_ was determined spectrophotometrically at 340 nm by measuring the increase in absorbance due to the production of NADH by the enzyme. The reaction mixture was analysed in a plastic cuvette at 50 °C and consisted of 2 mM NAD^+^, 40 µL of 0.38 mg/mL ALDH_Tt_, 10 mM potassium phosphate at a pH of 8, and 2 mM hexanal in a final reaction volume of 1.8 mL, unless otherwise stated. All ALDH_Tt_ reactions were continuously monitored for 2 min at 340 nm. The activity of ALDH_Tt_ was examined over a temperature range from 20 to 50 °C; higher temperatures were not utilised due to the volatility and flammability of the aldehyde substrates. All solutions (with the exception of the enzyme) were heated to the appropriate temperature in a water bath before use. Measurements were taken utilising a Cary60 UV-vis spectrophotometer equipped with a temperature controller. The activity of ALDH_Tt_ was also examined over a pH range of 2–10 using various buffers; pH 2 (10 mM potassium chloride-HCl buffer); 3−5 (10 mM citrate buffer); 6–8 (10 mM potassium phosphate); and 9–10 (10 mM Tris-HCl buffer).

### 2.7. Substrate Screening of Aldehydes 

Model aliphatic (hexanal) [30] and aromatic (benzaldehyde) substrates were tested for activity with the ALDH_Tt_ to first identify the substrate scope of the enzyme. Once conversion of both substrates was identified, in silico screening of aldehyde substrates was carried out, resulting in a library of 12 aliphatic, cyclic and aromatic aldehydes (Table S1) for use in enzymatic activity testing of the ALDH_Tt_ for determination of substrate specificity. The catalytic activity of ALDH_Tt_ was determined spectrophotometrically at 340 nm by measuring the increase in absorbance due to the production of NADH. The activity of ALDH_Tt_ for each of the 12 substrates was first analysed at 25 °C and further at 50 °C. The reaction mixture was analysed in a plastic cuvette, and consisted of 10 mM potassium phosphate buffer pH 8, 2 mM NAD^+^, 40 µL of 0.38 mg/mL ALDH_Tt_, and 2 mM of each substrate (1 mM for terephthalaldehyde due to solubility concerns) in a final reaction volume of 1.8 mL. All enzymatic assays were continuously monitored for 2 min.

### 2.8. ALDH_Tt_ Reduction Reaction

The action of ALDH_Tt_ for the reduction of carboxylic acids to aldehydes utilising NADH was investigated. The reduction reaction was determined spectrophotometrically by monitoring the decrease in absorbance at 340 nm due to the conversion of NADH to NAD^+^. The reaction mixture consisted of 10 mM potassium phosphate pH 8, 40 µL of 0.38 mg/mL ALDH_Tt,_ 150 µM NADH and either hexanoic acid (2 mM) or terephthalic acid (0.12 mM), with the reaction monitored for 5 min at 50 °C.

### 2.9. Determination of Kinetic Parameters 

Michaelis-Menten kinetics analysis was carried out for hexanal, benzaldehyde, and terephthalaldehyde, using the assay conditions outlined above at 25 and 50 °C varying substrate concentration. The ranges of concentrations used were as follows: 0.1–2 mM hexanal, 0.2–3.5 mM benzaldehyde, and 0.025–1 mM terephthalaldehyde.

Michaelis–Menten kinetics analysis for NAD^+^ was carried out using 2 mM hexanal at 25 and 50 °C. The NAD^+^ concentration varied from 1–200 µM.

### 2.10. HPLC Detection of Hexanoic Acid

To further demonstrate the catalytic mechanism of the ALDH_Tt_, the model substrate hexanal was utilised for conversion to hexanoic acid with direct detection of the carboxylic acid product via HPLC. An Agilent 1260 Infinity Series (Aglient Technologies, Palo Alto, Santa Clara, CA, USA) was used for HPLC analysis and the acquired data was processed with the Agilent OpenLAB CDS software. Chromatographic separations for the detection of hexanoic acid from ALDH_Tt_ enzymatic assays were carried out using an Agilent Microsorb-MV 100-5 C18 column (250 × 4.6 mm). The system was maintained at 30 °C with a run time of 10 min. The mobile phase which comprised of A: 10 mM potassium phosphate, phosphoric acid at a pH of 2.4, and B: HPLC gradient grade acetonitrile (Sigma Aldrich, Co. Wicklow, Ireland) (A:B, 60:40, v/v) was delivered to the column at a flow rate of 1 mL/min, which yielded a column back pressure of ~190 bar. Samples were filtered through 0.45 μm nylon filters and 60 μL injections were made. UV detection was conducted at a wavelength of 210 nm.

### 2.11. Esterase Activity

ALDH_Tt_ was purified in the absence of β-mercaptoethanol for esterase activity assessment in order to prevent artefactual catalysis of the ester. Following the nickel affinity chromatography step, the fractions containing ALDH_Tt_ were dialysed overnight against 50 mM Tris-HCl, 250 mM NaCl, pH 7.5, and subsequently loaded onto a HiLoad 16/60 Superdex 200 pg column pre-equilibrated with 50 mM Tris-HCl with a pH of 7.5, and 150 mM NaCl, snap frozen in liquid nitrogen and stored at −80 °C. To facilitate direct comparison of the esterase and dehydrogenase reaction rates, both activities were determined using protein samples purified without β-mercaptoethanol. The reactions were conducted in 10 mM potassium phosphate pH 8.0 at 25 °C unless otherwise stated. Two ester substrates were examined with ALDH_Tt_ at concentrations of 1 mM for *p*-nitrophenyl acetate (PNP-acetate) and 500 µM of *p*-nitrophenyl butyrate (PNP-butyrate). Standard solutions of PNP-acetate were prepared with acetone as a solvent to minimise spontaneous hydrolysis. Stock concentrations were selected so that the acetone concentration in the assay solutions did not exceed 0.1%. When direct comparison between the two activities was desired, a comparable acetone concentration was added to the standard assay system for dehydrogenase activity using hexanal. Esterase activity was monitored spectrophotometrically at 400 nm by measuring the increase in absorbance due to the production of the *p*-nitrophenoxide ion by the enzyme. All esterase data was corrected for the minor rate of spontaneous hydrolysis of substrate in the absence of enzyme (blank rate). A pH dependent extinction coefficient of 17 × 10^3^ M^−1^cm^−1^ was calculated via a calibration curve of *p*-nitrophenol in 10 mM potassium phosphate pH 8, 0.1% acetone (pKa = 7.1) (Figure S1).

## 3. Results

The 59 kDa his-tagged ALDH_Tt_ was overexpressed in *E. coli* BL21(DE3), and purified to apparent homogeneity using IMAC and gel filtration chromatography. Enzymatic activity assessments were carried out to determine the enzyme’s optimum pH and temperature for catalysis. In addition, 12 aldehyde substrates were tested for efficient conversion by the ALDH_Tt,_ with eight aliphatic, aromatic, and dialdehydes demonstrating catalytic activity. This sample pool of substrates demonstrated that the ALDH_Tt_ is not substrate specific, and indicates the capabilities of ALDH_Tt_ in terms of its substrates scope. ALDH_Tt_ was also examined for esterase activity demonstrating conversion of PNP-acetate and PNP-butyrate reliant on the absence of cofactor. Esterase activity occurred at a much lower rate than dehydrogenase activity, a common characteristic of ALDHs.

### 3.1. Protein Sequence Alignment 

BLAST search of the ALDH_Tt_ allowed for determination of its closest related homolog based on sequence identity. The putative ALDH from the thermophile *Thermus parvatiensis* with 529 amino acids resulted in a sequence identity of 97.92%, and is proposed to display a C-terminal extension characteristic of ALDH_Tt_. The highest related homologs (86–97% sequence identity) were all members of the *Thermus* genus, but none have been characterised to date. The well-characterised human ALDH1A3 returned a sequence identity of 39.03% in relation to ALDH_Tt_ (Figure S2), the highest identity of ALDHs with resolved structures. The closest mesophilic homolog was that of the SS-ALDH from *E. coli,* with a sequence identity of 34.30%. Interestingly, both human and mesophilic partners are tetramers similar to ALDH_Tt_; however, from the sequence alignment, neither contain a C-terminal extended tail (Figure S2). The ALDH_Tt_ is observed to contain an N-terminal deletion compared to ALDH1A3 but not SS-ALDH. 

### 3.2. Recombinant ALDH_Tt_ Expression and Purification

**Figure 1:**
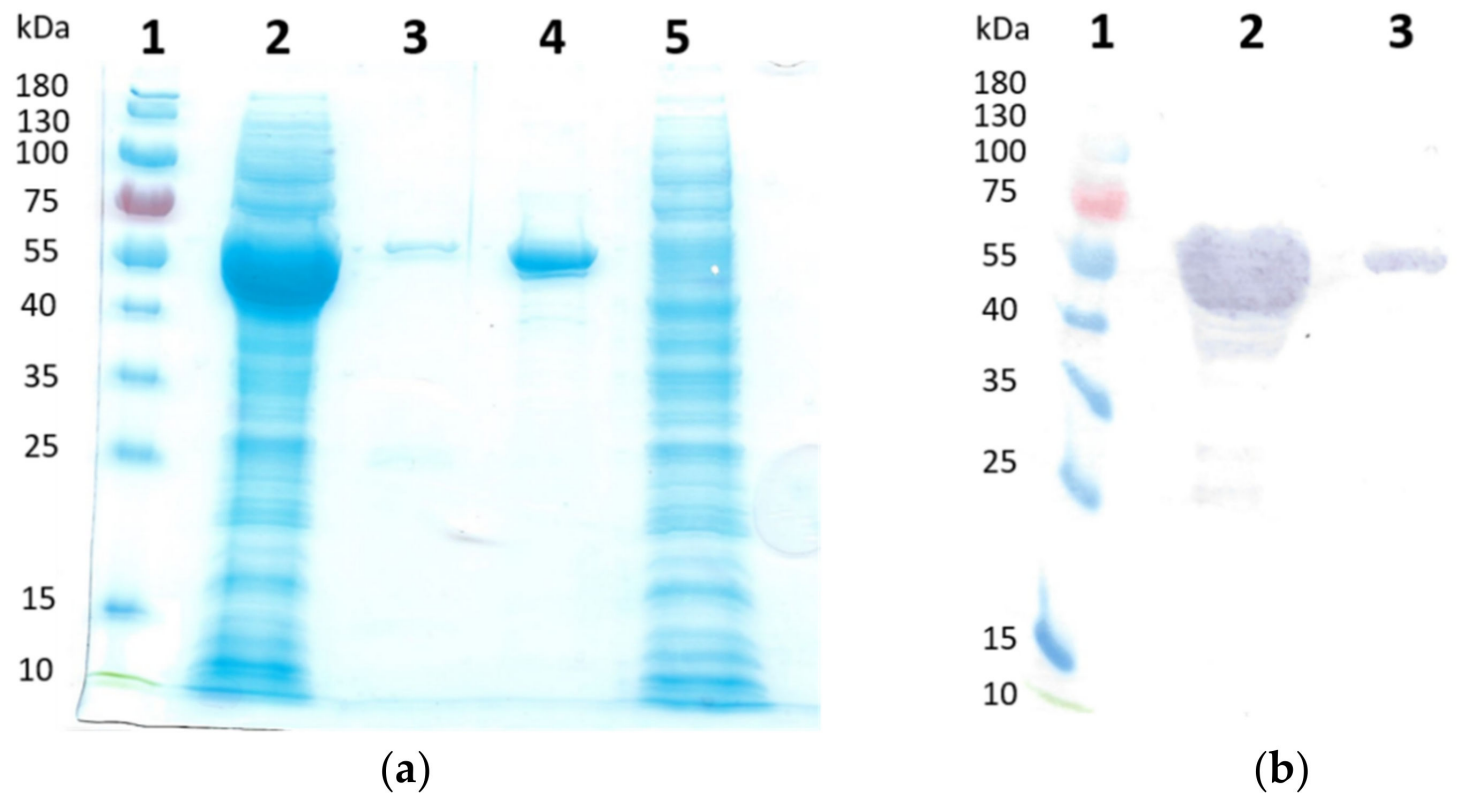
(**a**) SDS-PAGE of ALDH_Tt_ protein purification samples. Lane 1: PageRuler pre-stained protein ladder (ThermoFisher Scientific, Cork, Ireland), lane 2: *E. coli* BL21(DE3) cell lysate expressing ALDH_Tt_, lane 3: Ni affinity chromatography 200 mM imidazole elution, lane 4: purified ALDH_Tt_, lane 5: Ni affinity chromatography flow through. (**b**) Western blot of cell lysate expressing ALDH_Tt_ and purified protein following gel filtration chromatography. Lane 1: PageRuler pre-stained protein ladder (Thermofisher Scientific, Cork, Ireland), lane 2: *E. coli* BL21(DE3) cell lysate expressing ALDH_Tt,_ lane 3: purified ALDH_Tt_.

**Figure 2:**
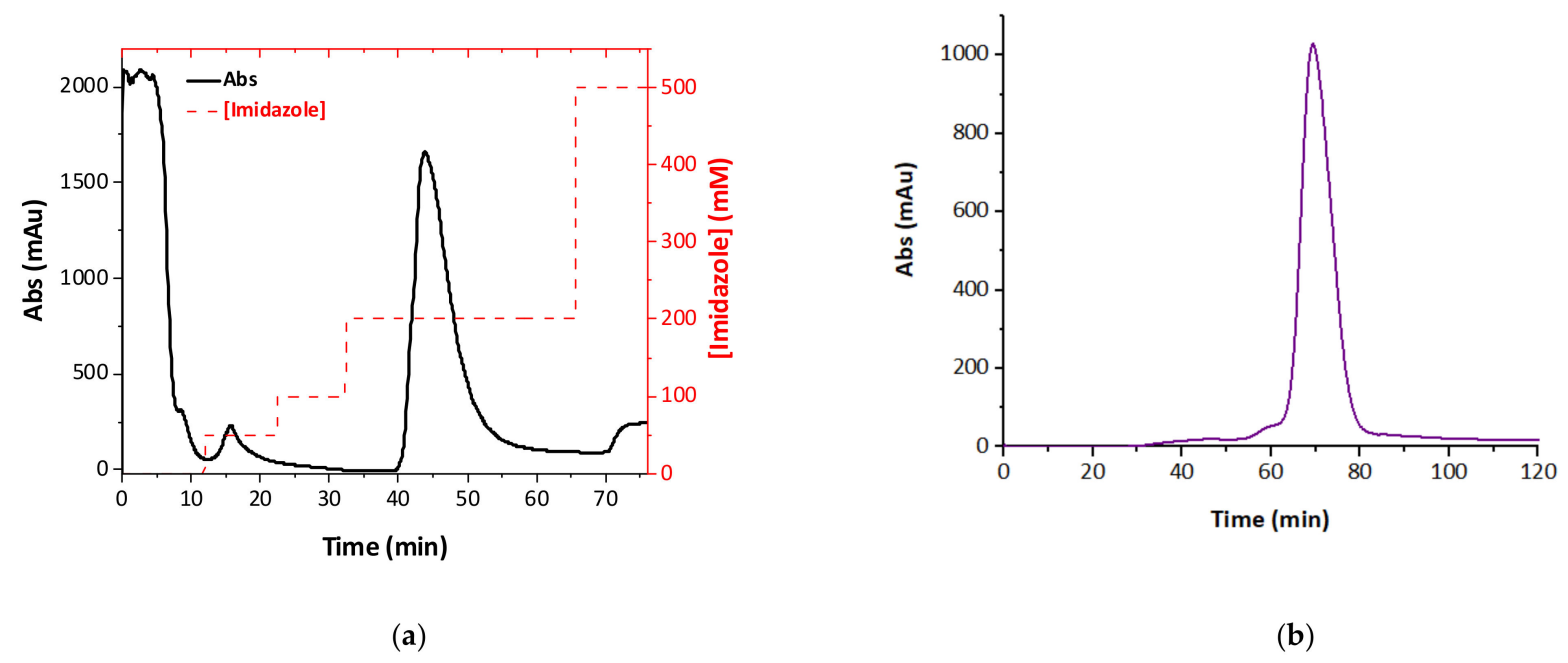
Elution profile of ALDH_Tt_ via Ni-affinity chromatography and gel filtration chromatography. Absorbance was monitored at 280 nm. (**a**) Chromatogram displaying purification of ALDH_Tt_ via Ni-affinity chromatography using a step gradient of imidazole from 50–500 mM. *E. coli* host proteins are eluted from 10–50 mM imidazole in the first two peaks, while ALDH_Tt_ is eluted at 200 mM imidazole in the third peak. (**b**) Chromatogram displaying a Gaussian peak of the apparent homogenous ALDH_Tt_ with elution at approximately 70 min using gel filtration chromatography at a flow rate of 1 mL/min.

Overexpression of ALDH_Tt_ was achieved in *E. coli* BL21(DE3) at 25 °C for 48 h (Figure 1a) with detection of the expressed his-tagged protein via Western blot, using anti-6x-his antibodies coupled with horse radish peroxidase for colorimetric detection using the substrate TMB (Figure 1b). The recombinant protein expressed in the cytosol of *E. coli* was purified to apparent homogeneity by lysis, heat treatment, immobilized metal affinity chromatography (IMAC), and gel filtration chromatography yielding approximately 15–20 mg/L of culture. During Ni affinity chromatography, ALDH_Tt_ eluted at 200 mM imidazole demonstrating substantial purity and efficient separation from host cell proteins (Figure 1a and Figure 2a). The gel filtration elution profile (Figure 2b) demonstrates apparent homogeneity of a tetrameric protein [30] with complete purity demonstrated on the SDS-PAGE (Figure 1a). Comparison of protein yield and purity was performed for the purification protocols with and without the heat treatment step. The fractions collected following Ni chromatography and gel filtration were of similar purity (Figure S3), irrespective of whether heat treatment was applied or omitted. However, the cell lysate of the heat-treated sample demonstrated higher purity with lower amounts of contaminating host proteins. Additionally, an approximate 40% decrease in protein yield occurred when the heat treatment step was removed from the purification protocol. Through the use of identical expression cultures, the protocol including heat treatment for 15 min at 65 °C yielded 13.42 mg/L, and when heat treatment was omitted, 8.18 mg/L was obtained.

### 3.3. Determination of ALDH_Tt_ Molecular Mass by MALDI-TOF

**Figure 3:**
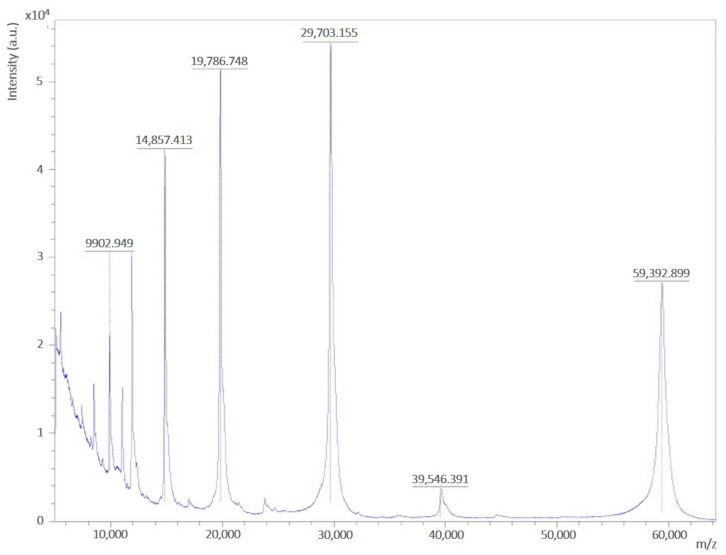
MALDI-TOF spectrum of recombinant ALDH_Tt_ utilising HCCA as the matrix, run in linear cationic 5–60 kDa mode, using 2500 shots.

The molecular mass of the 530 amino acid ALDH_Tt_ with attached his-tag estimated by SDS-PAGE (Figure 1a) agreed with the theoretical molecular mass deduced from the protein sequence (59.379 kDa), calculated using Expasy online molecular weight calculation tools (https://web.expasy.org accessed on 1 November 2021) and amino acid sequence (6FJX) from NCBI. MALDI-TOF analysis of the ALDH_Tt_ returned a molecular mass of 59.393 kDa (Figure 3) confirming the enzyme’s calculated molecular weight, and successful expression and purification of the full-length protein.

### 3.4. ALDH_Tt_ Enzymatic Activity Profiles 

**Figure 4:**
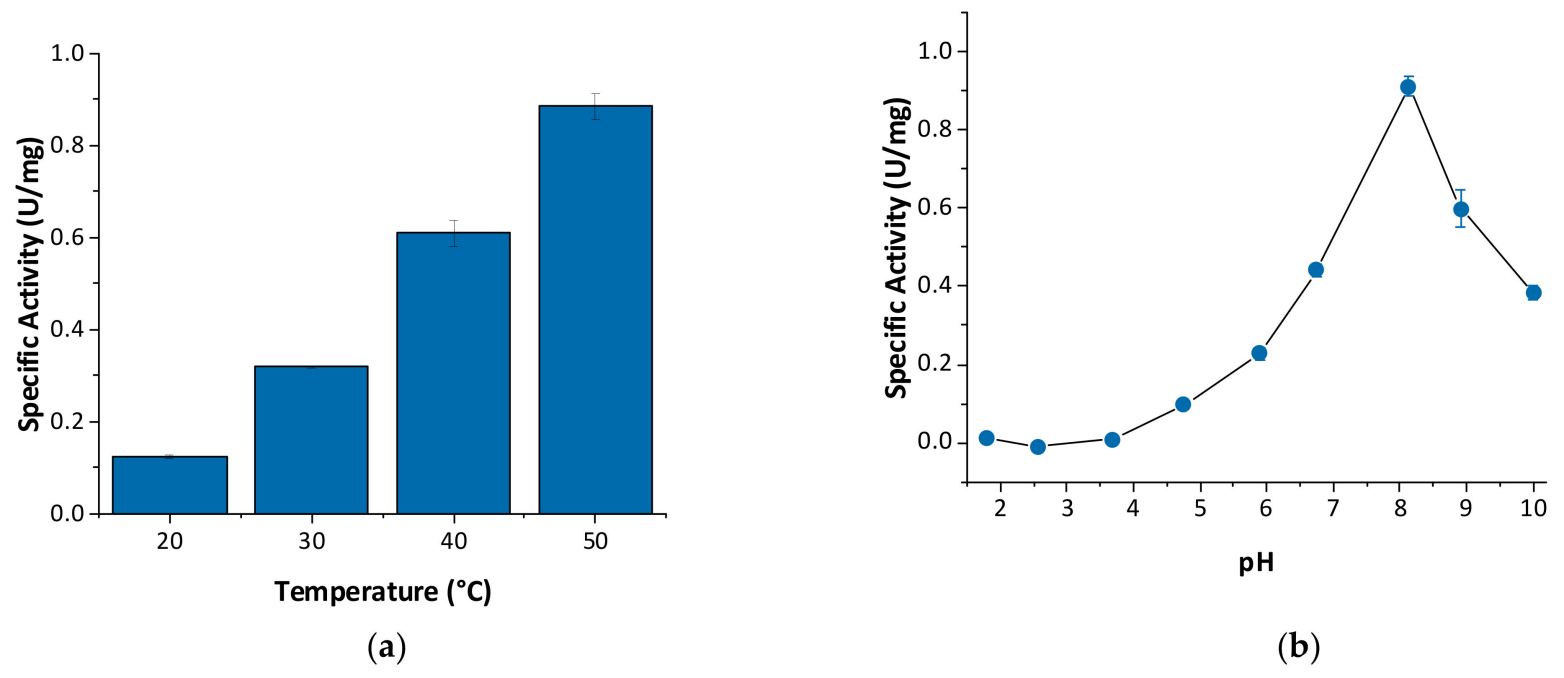
ALDH_Tt_ enzymatic activity profiles utilising NAD^+^ as cofactor and hexanal as substrate (**a**) Plot of activity of ALDH_Tt_ as a function of temperature in the range 20–50 °C in 10 mM potassium phosphate pH 8. (**b**) Plot of activity of ALDH_Tt_ as a function of pH at 50 °C. Optimum conditions were determined as pH 8 at 50 °C.

The optimum operation conditions of the ALDH_Tt_ were explored, over a range of temperatures and pHs using the model substrate hexanal with NAD^+^ as cofactor. Activity at 20 °C was 7.2-fold less than that at 50 °C (0.12 ± 0.003 and 0.86 ± 0.03 U/mg, respectively) (Figure 4a). The operational pH range of the enzyme was also examined, with an optimal pH for use of 8 with a specific activity of 0.91 ± 0.03 U/mg (Figure 4b). The activity in basic conditions was more favourable than the acidic ones. Over the pH range of 2–5, the activity of the enzyme was miniscule. Overall, 10% of maximum activity was observed at pH 5, while at neutral (6–7) and basic (9–10) pH the activity was 37 and 54%, respectively, of that at pH 8. 

**Figure 5:**
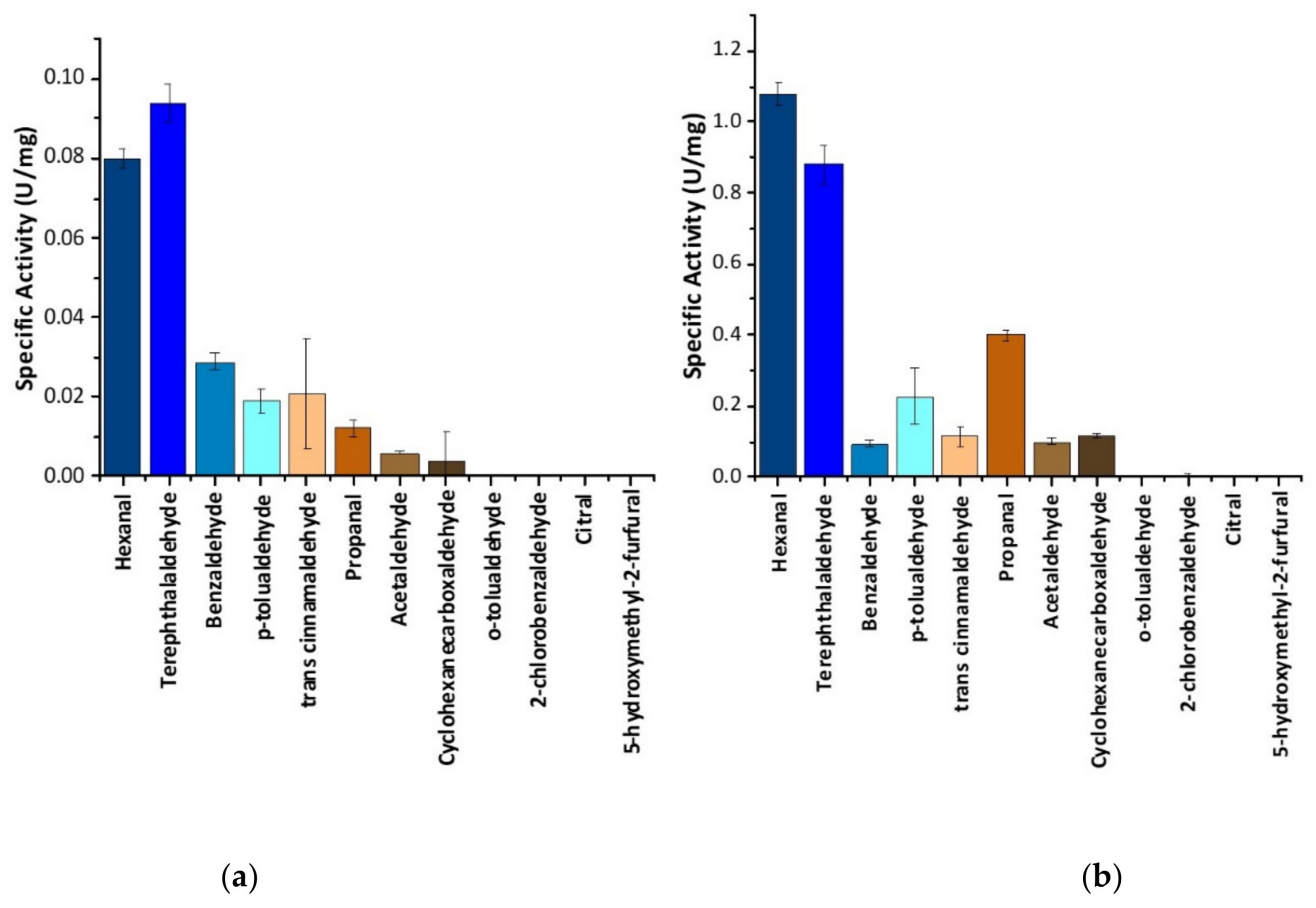
ALDH_Tt_ substrate screening and specificity utilising a range of aliphatic, aromatic, and cyclic aldehydes. Specific activity is demonstrated at (**a**) 25 °C and (**b**) 50 °C, displaying the thermophilic nature and broad substrate scope of the enzyme.

ALDH_Tt_ has recently been reported [30] to possess the highest catalytic activity for the substrate hexanal (1.08 ± 0.03 U/mg). Through screening of a range of aldehydes, it was determined that ALDH_Tt_ can oxidise aliphatic, aromatic, and dialdehydes, specifically, hexanal, propanal, acetaldehyde, benzaldehyde, *p*-tolualdehyde, *trans*-cinnamaldehyde, and terephthalaldehyde, at reasonable rates of oxidation (Figure 5 and Table S1). Similar activity, with respect to hexanal at 50 °C, was observed using the synthetic dialdehyde, terephthalaldehyde (0.88 ± 0.05 U/mg). Other substrates such as cyclic cyclohexanecarboxaldehyde, 5-hydroxymethyl-2-furfural, and citral displayed no catalytic rate at 25 °C, similar to *ortho*-substituted benzaldehydes, *o*-tolualdehyde, and 2-chlorobenzaldehyde. Substrates which demonstrated conversion at 25 °C showed increased specific activity at 50 °C (approximately 10-fold (Figure 5b)). The substrate cyclohexanecarboxaldehyde that displayed minor and inconsistent catalytic activity at 25 °C demonstrated a boost in activity at 50 °C (0.11 ± 0.008 U/mg). Moreover, 2-chlorobenzaldehyde showed minor activity at 50 °C; however, with large experimental error (0.005 ± 0.004 U/mg). Additionally, ALDH_Tt_ dehydrogenase activity is irreversible. No catalytic activity was detected for conversion of hexanoic acid nor terephthalalic acid utilising NADH as cofactor. 

ALDH_Tt_ follows Michaelis–Menten type mechanism, and the kinetic parameters (K_M_, V_max_ and k_cat_) for hexanal, benzaldehyde, and terephthalaldehyde were calculated using NAD^+^ as cofactor at both 25 and 50 °C. (Table 1 and Figure S4). The K_M_ remained similar at both temperatures across the substrates, with an increase in V_max_ and k_cat_ observed at 50 °C, as expected. Comparison of the substrates at 25 and 50 °C demonstrated that terephthalaldehyde displayed the lowest K_M_ (0.11 and 0.38 mM), followed by hexanal (1.08 and 0.99 mM) and benzaldehyde (1.41 and 1.52 mM). The K_cat_ values follow a similar trend with the fastest rate demonstrated by terephthalaldehyde (1.05 and 12.08 s^−1^), followed by hexanal (0.51 and 5.71 s^−1^), with benzaldehyde displaying the slowest turnover number (0.19 and 1.46 s^−1^) (first value stated in brackets is 25 °C, second is 50 °C). The K_M_ for the cofactor, NAD^+^, of 4.7 µM (25 °C) and 9.2 µM (50 °C), was significantly lower than the substrates trialled, demonstrating increased affinity for the cofactor (Figure S5).

Previous determination of enzymatic conversion of aldehyde and NAD^+^ to carboxylic acid and NADH by the ALDH_Tt_ was determined by following NADH production at 340 nm via UV-Vis spectrophotometry. Here, detection of the carboxylic acid product by HPLC has been performed, to further confirm the ALDH_Tt_ reaction mechanism and products formed. A 2 h enzymatic assay of ALDH_Tt_ with hexanal as substrate was run at 50 °C monitoring production of NADH over time (Figure S6). The resultant product was analysed via HPLC. The ALDH_Tt_ assay contains a number of components, most notably NAD^+^, NADH, hexanal, and hexanoic acid. Good separation of these compounds from the hexanoic acid product was achieved allowing for detection of the product. NAD^+^ and NADH had a retention time of approximately 2–3 min, hexanal at 4.44 min, and hexanoic acid at 6.86 min. From the 2 h ALDH_Tt_ assay, hexanoic acid was detected in the assay mixture (Figure S7) confirming dehydrogenase activity by ALDH_Tt_.

**Figure 6:**
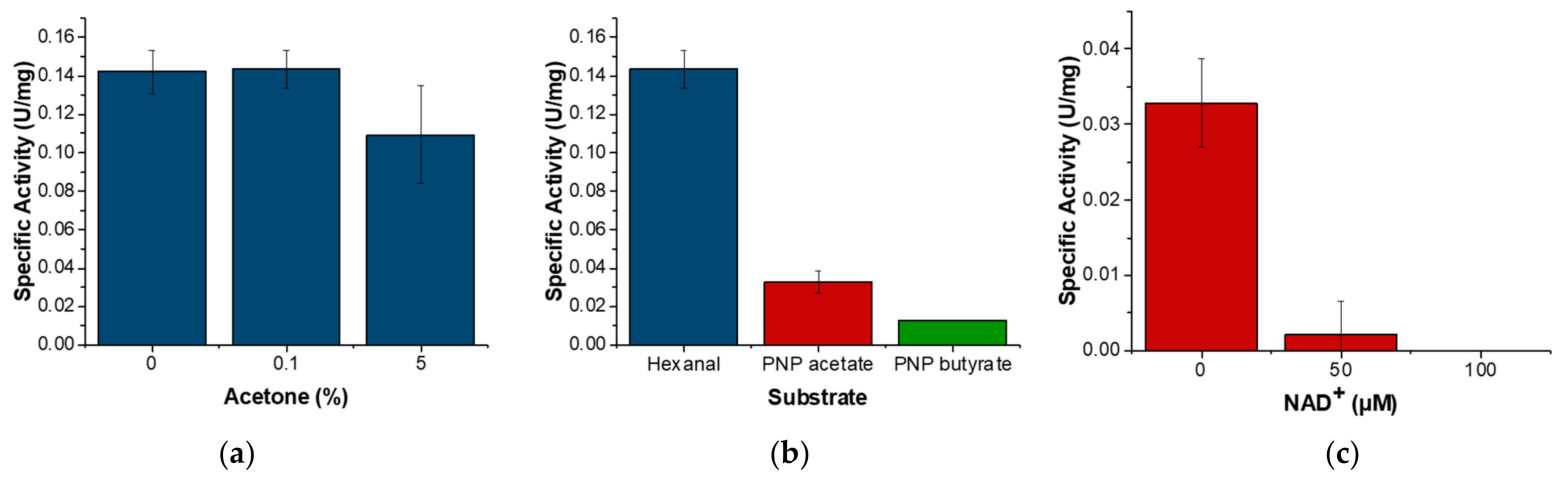
Dehydrogenase and esterase activity of ALDH_Tt_ at 25 °C purified in the absence of β-mercaptoethanol (**a**) dehydrogenase activity using hexanal in the presence of acetone, (**b**) comparison of specific activity of dehydrogenase and esterase activity using different esters, (**c**) esterase activity using PNP-acetate in the presence of low concentrations of NAD^+^.

ALDH_Tt_ is capable of acting as an esterase and can catalyse the conversion of *p*-nitrophenyl esters to a carboxylic acid and alcohol. The ALDH_Tt_ was purified in the absence of β-mercaptoethanol, which would typically be present in 5 mM in the final protein formulation. Firstly, dehydrogenase activity of the ALDH_Tt_ was tested to ensure the enzyme was catalytically active and stable while omitting β-mercaptoethanol. Specific activity, for hexanal at 25 °C, of 0.14 ± 0.01 U/mg was demonstrated (Figure 6a). The model ester substrate used in esterase activity for ALDH enzymes in the literature is PNP-acetate [9,10,22,45]; however, its use requires preparation in acetone to avoid spontaneous hydrolysis. The tolerance of ALDH_Tt_ dehydrogenase activity to increased levels of acetone (0.1 and 5%) was examined. The rate of oxidation of hexanal by ALDH_Tt_ was conserved in 0.1% acetone; however, a decrease of approx. 23% was observed in 5% acetone (Figure 6a), requiring the use of stock solutions of PNP-acetate in acetone at concentrations, which, upon addition, would result in 0.1% acetone in the final reaction volume so as not to disrupt enzyme activity. ALDH_Tt_ is active for PNP-acetate, resulting in a specific activity of 0.033 ± 0.006 U/mg. In addition, activity was demonstrated for PNP-butyrate, but at the lower rate of 0.013 ± 0.0002 U/mg (Table S2). Compared to dehydrogenase activity for hexanal, esterase activity with both esters trialled is significantly slower (Figure 6b), 22.9 and 8.8% residual activity for PNP-acetate and PNP-butyrate, respectively. Upon calculation of esterase activity, the small background rate of spontaneous hydrolysis of the ester (blank rate) (Figure S8) was removed from the enzymatic rate. Since ALDH_Tt_ is thermophilic, esterase activity for PNP-acetate was further analysed at 50 °C, but spontaneous hydrolysis of the substrate at the increased temperature was too high to monitor enzymatic activity (Figure S9), displaying a slope of 0.21, comparable to the enzyme samples. Esterase activity does not require the addition of the NAD^+^ cofactor that dehydrogenase activity by ALDH_Tt_ demands. Upon addition of low concentrations of NAD^+^ (50 µM), esterase activity for PNP-acetate was greatly diminished. At levels of 100 µM NAD^+^ no esterase activity was evident (Figure 6c).

## 4. Discussion

In 1999, when the focus of ALDH studies was primarily eukaryotic models, a systematic classification system based on protein sequence identity was adopted [46]. This stated that proteins with a sequence identity of above 40% were members of the same family, while enzymes with a sequence identity above 60% were members of the same subfamily. Recently, ALDH study has delved into prokaryotic models, demonstrating difficulty in obtaining a sequence identity above 40%, due to the newly recognised diversity of ALDHs as well as the plethora of prokaryotic species. For example, study of the *Pseudomonas* genus alone lead to the identification of 42 different ALDH classes [31]. BLAST search of ALDH_Tt_ resulted in the highest sequence identity for ALDH1A3, while both enzymes display strikingly similar biochemical characteristics [47]. Both ALDH_Tt_ and ALDH1A3 are NAD^+^ favouring, soluble enzymes, whose catalytic rate is reduced through use of NADP^+^ cofactor. ALDH1A3 uses the primary substrate retinaldehyde for biosynthesis of retinoic acid. Interestingly, when ALDH1A3 was analysed for activity using non-retinoid aldehydes, hexanal proved to be the best substrate with the highest catalytic efficiency [22]. However, while ALDH1A3 demonstrates a lower K_M_ for hexanal (6.0 µM) than NAD^+^ (130 µM) [22], ALDH_Tt_ displays the inverse. ALDH1A3 has a much higher affinity for the hexanal substrate than ALDH_Tt_, whereas ALDH_Tt_ shows higher affinity for the cofactor. ALDH1A3 also demonstrated esterase activity for PNP-acetate, alike to ALDH_Tt_.

Across ALDHs, substrate specificity has been related to the shape and geometry of the substrate entry channel of the enzyme, linked to three key amino acids at distinct positions, termed the “mouth” (136), “neck” (471), and “bottom” (315) (human ALDH1A3 numbering) [5,22,48]. ALDH1A3 and ALDH_Tt_ possess a conserved Thr at the “bottom” residue, and show a conservative substitution of hydrophobic residues at the “neck” (Leu to Ala respectively). This conservative substitution is demonstrated across human ALDH1 family members [22]. A significant exchange of Gly to Glu at the “mouth” in ALDH1A3 vs ALDH_Tt_ is evident. The mouth residue typically carries out a size selection function, for example human ALDH2 with the preferred substrate acetaldehyde possesses a bulky Met at this position, while ALDH1A3 using retinaldehyde harbours a Gly [5,48,49,50]. ALDH_Tt_ with Glu at the “mouth” position might relate to optimal substrates being smaller than retinaldehyde, but larger than acetaldehyde, a trend which was adhered to experimentally with the trialled substrates, e.g., hexanal, *trans*-cinnamaldehyde, and terephthalaldehyde.

Two thermophilic ALDHs with resolved structures, ALDH_Pp_ (PDB: 4JZ6) and ALDH from *Thermoproteus tenax* (ALDH_Ttx_) (PDB: 1UXT), acquire 30.75 and 29.23% sequence identity, respectively, compared to ALDH_Tt_ [38,51]. Both are NAD^+^ favouring enzymes, with ALDH_Pp_ acting as a dimer and ALDH_Ttx_ as a tetramer. ALDH_Pp_ displays broad substrate specificity with the ability to process aliphatic and aromatic aldehydes at high catalytic efficiencies, including hexanal and benzaldehyde. ALDH_Ttx_, however, is specific for glyceraldehyde-3-phosphate. Through analysis of a range of thermophilic ALDHs, it was found that they are quite diverse in oligomeric state and substrate specificity. ALDH_Gtv_ exists as an octamer, with ALDH_Gtd_ displaying a tetrameric formation with the possibility of octamers formed via dimerization of tetramers [35,39]. Overall, thermophilic ALDHs had a high preference for NAD^+^ [35,38,39,51,52]. An exception is the archaeal ALDH from *Sulfolobus solfataricus* (ALDH_Ss_) favouring NADP^+^ [53]. The ALDH from *Sulfolobus tokodaii* (ALDH_St_) also demonstrated esterase activity for PNP-acetate [52], displaying that thermophilic counterparts also display dual enzymatic activity (discussed further below).

Interestingly, ALDH_Tt_ was first co-purified as a contaminant during native *caa_3_*-type cytochrome oxidase isolation from *T. thermophilus* [44]. This is peculiar as, in mammals, ALDH and cytochrome oxidases are linked to the biosynthesis of retinoic acid. In vivo retinoic acid synthesis starts from β-carotene or all-trans-retinol to produce retinaldehyde, which is then converted to all-trans retinoic acid via ALDH1 isozymes; this is further processed to form 4-hydroxy-retinoic acid utilising cytochrome oxidases. This highlights a biological link between the two enzymes and the possibility of a similar mechanism within *T. thermophilus*. Additionally, the sequence relation between ALDH1A3 and ALDH_Tt_ further highlights this possibility as ALDH1A3′s primary substrate is retinaldehyde. However, retinoid signalling mechanisms are generally considered to be of animal origin, and to date it has only been characterised in animals. More recently, retinoic acid was identified within prokaryotic models, including *Bacillus cereus* [37] and cyanobacteria [36], exhibiting a potential biological role of retinoic acid in prokaryotes. It was hypothesised that perhaps the retinoic acid signalling pathway first existed in cyanobacteria and now exists in humans through a lateral gene transfer event of ALDH and CYP120 [36,54], which was also hypothesised for other proteins [55,56,57]. The cyanobacteria *Chlorogloeopsis fritschii* PCC 6912 possesses three orthologs of the enzymes responsible for the conversion of β-carotene to retinoic acid in the signalling pathway recognised in animals. Interestingly, the ALDH was recognised to convert retinaldehyde to retinoic acid suggesting evidence of the use of the pathway in prokaryotic models, and one of the possible functional roles of ALDH_Tt_.

Recombinant expression and purification of ALDH_Tt_ proved an efficient method for the production of the full-length enzyme displaying high purity, while also obtaining high production yields. Heat precipitation protocols have been regularly incorporated as a selective purification step for thermostable proteins expressed in mesophilic hosts [53,58,59,60,61], and has proven effective with other *T. thermophilus* proteins expressed in *E. coli,* including other dehydrogenases and the ALDH family member 1-pyrroline-5-carboxylate dehydrogenase (P5CDH_Tt_) [61,62,63]. The heat treatment step of purification of alcohol dehydrogenase, from *T. thermophilus,* at 75 °C for 15 min was deemed the most efficient step, enriching the enzyme 11-fold, and resulting in 47% yield of a homogeneous protein [61]. Similarly, isocitrate dehydrogenase was purified by heating at 70 °C for 20 min, resulting in 80% purification and increased yields [63] (exact figures not stated), compared to previous reports utilising purification mechanisms from native *T. thermophilus*. Heat treatment is a promising step to include in a purification protocol, as it does not involve any chemical reagents while also denaturing and aggregating host proteins. In addition, cellular proteases are often denatured, removing their threat to the desired protein product, and thus increasing product yield. Previous studies have demonstrated that addition of heat treatment steps to purification protocols can allow for increased yield and purity [64,65]. Heat treatment can further facilitate release of protein product from undisrupted *E. coli* cells increasing the overall yield of the process [66]. However, the process must be optimised so as not to denature the protein of interest. In this protocol, for ALDH_Tt_ purification, the heat treatment step occurs before the removal of cell debris, and acts not only as a method for inactivation of host cell proteins, but also as an additional cell disrupting mechanism. A previous report outlined how increased purity and yield of recombinant thermophilic catalase from *Bacillus sp.* expressed in *E. coli* was achieved via heat treatment purification at 65 °C for 2 h, resulting in a three-fold increase in specific activity of the cell supernatant [59]. Three temperatures were trialled for purification, 55, 60, and 65 °C, demonstrating increased catalase purity with increased temperature. However, when 70 °C was utilised, catalase was denatured and precipitated, outlining the importance of optimisation. This was also highlighted through purification of ribose-5-phosphate isomerase from *Thermotoga maritima* where buffer choice, temperature, and treatment time were extensively optimised to achieve high purity (95%) and high yields (44.8 mg/L) [58]. Previous reports have also discussed the efficient use of heat treatment purification for mesophilic proteins, such as hepatitis B core antigen and unstructured regions of the proteins epsin 1 and AP180, with an increase in yield in the range of 18–106% [64,65]. 

As a thermophilic enzyme, the specific activity of ALDH_Tt_, is strongly affected by temperature. It is expected that the specific activity of ALDH_Tt_ would increase further at elevated temperatures up to 80 °C, given the optimum growth conditions of *T. thermophilus* [67,68]. However, temperatures exceeding 50 °C were not utilised due to volatility and flammability of the aldehyde substrates. In addition, the operable pH range demonstrated mimics the optimal growth conditions of the bacterium, favouring neutral pHs with optimum enzymatic activity achieved at pH 8. Investigation of other enzymes from *T. thermophilus* demonstrated similar trends with respect to effect of temperature and pH [6,61,69]. P5CDH_Tt_ was also assayed at 50 °C using the substrate Δ^1^-pyrroline-5-carboxylate with enzymatic activity observed in the pH range of 6–10, with a broad optimum from 7–10 [6]. An alcohol dehydrogenase [61] and glutamate racemase [69] from *T. thermophilus* showed highest catalytic activity at a pH between 9–10, with little or no activity present in acidic conditions. The enzymes demonstrated their highest specific activity at 73 and 85 °C, respectively. Thermophilic ALDHs tend to work best under neutral to basic conditions at temperatures from 50–80 °C, as demonstrated by P5CDH_Tt_ above. Other examples include, ALDH_Gtv_, which works optimally at 55 °C, pH 10 [35], ALDH_Pp_ at 60 °C, pH 8.5 [38], and ALDH_Gtd_ at 60 °C, pH 8 [39]. Interestingly, the former two of these ALDHs were not stable once a temperature of 70 °C was reached, with ALDH_Gtd_ displaying 56% residual activity following incubation at 70 °C for 1 h. In comparison ALDH_Ss_ was assayed at 70 °C, pH 7 [53] and ALDH_St_ at 80 °C, pH 8 [52]. 

Many ALDH enzymes are reported as being non-specific for substrate, demonstrating catalytic activity with a range of natural and synthetic aldehydes, including thermophilic members [16,22,38,39,40,52,70,71]. ALDH_Tt_ could convert aliphatic and aromatic aldehydes, *para*-substituted benzaldehydes could be efficiently converted by ALDH_Tt_, while *ortho*-substituted benzaldehydes demonstrated no catalytic activity. Previous reports outline that *ortho*-substituted benzaldehydes can result in reduced ALDH activity due to steric hindrance effects around the aldehyde group, particularly when the associated substitution group is bulky [16,40]. Thermophilic ALDH_Gtd_ could process 2-chlorobenzaldehyde and *o*-phthalaldehyde, but at low residual activities, compared to formaldehyde of 28.4 and 10.8%, respectively [39], while ALDH_Pp_ could also convert *ortho*-substituted benzaldehydes [38]. The thermophilic enzymatic action of the ALDH_Tt_ at 50 °C was maintained with the selection of substrates, highlighting that substrate specificity is not dependent on temperature.

ALDH_Tt_’s broad substrate specificity coupled with its increased specific activity at elevated temperatures makes it an attractive target for industrial synthesis of carboxylic acids via biocatalytic mechanisms. Its thermostability will allow for its use in biocatalysis at temperatures where mesophilic counterparts may be unstable or become denatured. The substrate scope of ALDH_Tt_ provides a range of interesting potential carboxylic acid products, most notably terephthalic acid and *p*-toluic acid, which are used as a precursor in polyethylene terephthalate (PET) plastic manufacture and as an intermediate in terephthalic acid and polymer synthesis, respectively [72,73].

It was previously demonstrated that ALDH_Tt_ has a significant preference for NAD^+^ cofactor rather than NADP^+^_,_ displaying 33.4 % residual activity when NADP^+^ is used with hexanal at 50 °C [30], a trend mostly adhered to by thermophilic ALDHs. As reported for other ALDHs [47,53,74] the K_M_ for NAD^+^ is significantly lower than that of the substrates analysed. The K_M_ for terephthalaldehyde was significantly lower than the other two substrates, possibly indicative of its dialdehyde nature and the availability of extra aldehyde groups for binding to the ALDH_Tt_. In contrast, benzaldehyde with only one aldehyde group but a similar structure has a higher K_M_ than terephthalaldehyde. Similar results were obtained using ALDH_Gc_ [16], where the activity of the enzyme increased 3.6-fold when using the dialdehyde terephthalaldehyde versus benzaldehyde. Additionally, when a similar substrate, terephthalaldehylic acid, consisting of one aldehyde group and one carboxylic acid group, was used by ALDH_Gc_, it decreased by 14.3- and 51.0-fold with respect to benzaldehyde and terephthalaldehyde. This further highlights the use of the two aldehyde groups on dialdehydes by ALDHs for decreased K_M_, and increased specific activity. Furthermore, when ALDH_Gc_ utilised dialdehydes with *ortho*-substituted Br, its specific activity was comparable to benzaldehyde (113–115% residual activity) due to the availability of the second aldehyde group for processing, whereas *o*-bromobenzaldehyde only demonstrated 8% residual activity compared to benzaldehyde. However, ALDH_Gc_ demonstrated miniscule residual activity (˂1%) for *o*-phthalaldehyde, constructed by a benzaldehyde with an *ortho*-substitution of the second aldehyde group. Here, the availability of both aldehyde groups is occluded due to steric hindrance, resulting in minor specific activity. 

ALDH_Tt_ has dual dehydrogenase and esterase activity and can act independently on aldehyde or *p*-nitrophenyl ester substrates. The esterase activity of ALDHs was first realised in human [75] and horse liver [9] ALDH, and there is evidence that both activities take place within one active site [12]. An array of ALDHs have demonstrated esterase activity for the substrate PNP-acetate to form acetic acid and *p*-nitrophenol, including prokaryotic models [2,22,52,76,77]. However, the rate of esterase activity of ALDHs is often much lower than their associated dehydrogenase activity. A residual activity of 1–15% is common [76,78]. ALDH_Tt_ displayed esterase activity for the conversion of two ester substrates, PNP-acetate and PNP-butyrate, without requiring the addition of NAD^+^ cofactor. Throughout the literature, it is highlighted that the addition of NAD^+^ can either accelerate the ALDH esterase reaction or inhibit it, depending on the isozyme in question [9,10,15,78,79]. Low concentrations of cofactor activated the esterase reaction of sheep ALDH1; however, concentrations above 200 µM slowed the reaction. A study demonstrated that human mitochondrial ALDH2′s esterase action is accelerated by low concentrations of NAD^+^ (2.5 fold with 200 µM), whereas the same concentrations (50–200 µM) nearly abolished human ALDH3 esterase activity [15]. This likely highlights that ALDH_Tt_ acquires a rate-limiting step characteristic of human ALDH3 enzymes for the esterase reaction; however, its closest related homolog by sequence identity was the human ALDH1A3. In the presence of 500 µM NAD^+^ ALDH1A3 exhibited esterase activity for PNP-acetate [22]. Again, this suggests that prokaryotic ALDH models are unique in their own right, and might contain a cross-over of characteristics from distinct mammalian classes and models. Interestingly, the thermophilic ALDH_St_ displayed esterase activity for PNP-acetate, and was also inhibited by NAD^+^ [52]. Esterase activity by this enzyme was reduced by 90% in the presence of 500 µM NAD^+^ and activity was abolished at a concentration of 1 mM. 

## 5. Conclusions

In summary, it can be concluded that the tetrameric ALDH_Tt_ from *T. thermophilus* is a bifunctional enzyme with inherent dehydrogenase and esterase activity, while being non-substrate specific in relation to both reactions. The ALDH_Tt_ can catalyse the oxidation of aliphatic, aromatic, and dialdehydes, most notably hexanal and terephthalaldehyde. Biochemical characterisation of the enzyme in terms of substrate scope helps understand possible biological mechanisms, such as the detoxification of certain aldehyde substrates or retinoic acid biosynthesis via association with cytochrome oxidase. Characterisation of the lesser studied prokaryotic ALDH models is important to broaden the understanding of this ever-growing superfamily. Additionally, the thermophilic nature of the ALDH_Tt_, working in the range of 20–50 °C, coupled with its broad substrate scope, allows for application of this enzyme in biocatalytic mechanisms. Possible applications relate to the oxidation of aldehydes to synthesise carboxylic acids chemoselectively, or even the hydrolysis of esters for production of desirable alcohols. However, the reduced catalytic rate of the latter needs to be considered.

## Figures and Tables

**Table 1:** Kinetic parameters for ALDH_Tt_ using hexanal, terephthalaldehyde and benzaldehyde with NAD^+^.

Substrate	°C	K_M_ (mM)	V_max_ (µmol/min)	k_cat_ (s^−1^)
Hexanal	25 °C	1.08 ± 0.46	0.0021 ± 0.0006	0.51 ± 0.15
	50 °C	0.99 ± 0.24	0.027 ± 0.0043	5.71 ± 0.64
Terephthalaldehyde	25 °C	0.11 ± 0.067	0.0041 ± 0.001	1.05 ± 0.27
	50 °C	0.38 ± 0.11	0.047 ± 0.010	12.08 ± 2.50
Benzaldehyde	25 °C	1.41 ± 1.15	0.0008 ± 0.0003	0.19 ± 0.09
	50 °C	1.52 ± 0.51	0.0057 ± 0.001	1.46 ± 0.29

## Data Availability

Data is contained within the article or Supplementary Materials.

## Supplementary Materials

The following are available online at https://www.mdpi.com/article/10.3390/cells10123535/s1, Figure S1: Calibration curve of p-nitrophenol in 10 mM potassium phosphate pH 8, 0.1% acetone, Figure S2: Protein sequence alignment of ALDH_Tt_ (PDB: 6FJX) and (A) ALDH1A3 (PDB: 5FHZ) and (B) succinate semialdehyde dehydrogenase (SS-ALDH) from *E. coli* (PDB: 3JZ4), Figure S3: SDS-PAGE of ALDH_Tt_ expression and purification fractions omitting the heat treatment step. Lane 1: PageRuler pre-stained protein ladder (ThermoFisher), lane 2: *E. coli* BL21(DE3) cell lysate expressing ALDH_Tt_, lane 3: Ni affinity chromatography 200 mM imidazole elution, lane 4: purified ALDH_Tt_,, lane 5: Ni affinity chromatography flow through, Figure S4: Michaelis–Menten kinetic plots for ALDH_Tt_ with different substrates, (A) hexanal 25 °C, (B) hexanal 50 °C, (C) benzaldehyde 25 °C, (D) benzaldehyde 50 °C, (E) terephthalaldehyde 25 °C, (F) terephthalaldehyde 50 °C, Figure S5: Michaelis–Menten kinetics plot varying [NAD+] for ALDH_Tt_ using hexanal at (A) 25 °C and (B) 50 °C, Figure S6: ALDH_Tt_ reaction course over 2 h using hexanal with NADH production monitored at 340 nm, Figure S7: HPLC chromatogram for the detection of hexanoic acid from 2 h ALDH_Tt_ assay with a retention time of 6.86 min, Figure S8: Absorbance vs. time for esterase activity monitored at 400 nm at 25 °C using (A) PNP-acetate and (B) PNP-butyrate, Figure S9: Absorbance vs. time monitored at 400 nm for esterase activity at 50 °C using PNP-acetate, Table S1: Aldehyde substrates used for catalysis by ALDH_Tt_ highlighting their associated specific activity, Table S2: Esterase substrates catalysed by ALDH_Tt_ highlighting their associated specific activity.
