# Supplementary material for: Study of ALDH from Thermus thermophilus—Expression, Purification and Characterisation of the Non-Substrate Specific, Thermophilic Enzyme Displaying Both Dehydrogenase and Esterase Activity
Source: Cells. 2021 Dec 14;10(12):3535. doi: 10.3390/cells10123535 (PMC8699947; doi:10.3390/cells10123535)
Supplement: Supplementary file 1 [file cells-10-03535-s001.zip › cells-1483948 - sup - 12.23.pdf]

Supplementary Material

Study of ALDH from *Thermus thermophilus* – expression, purification and characterisation of the non-substrate specific, thermophilic enzyme displaying both dehydrogenase and esterase activity

Kim Shortall [1], Edel Durack [2] Edmond Magner [1] and Tewfik Soulimane [1,*]

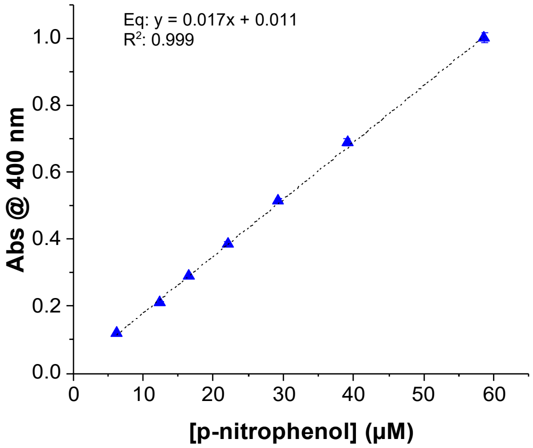

Figure S1: Calibration curve of p-nitrophenol in 10 mM potassium phosphate pH 8, 0.1 % acetone.

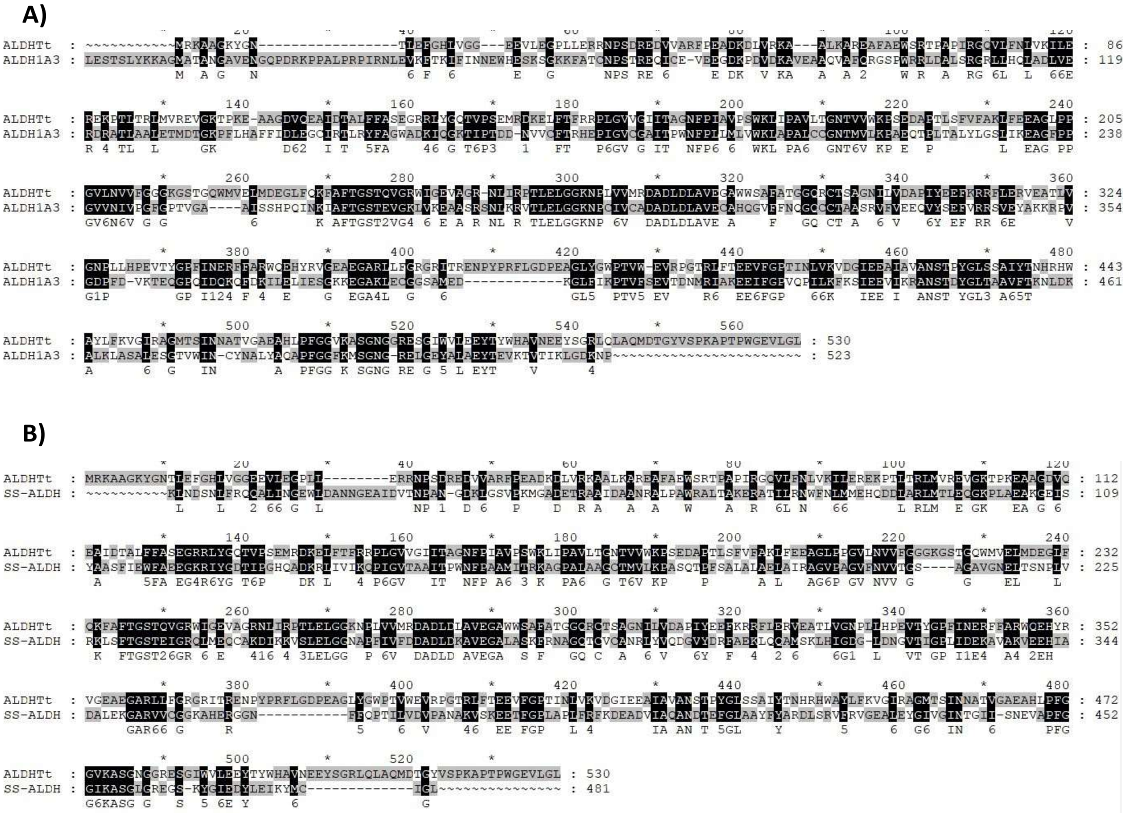

Figure S2: Protein sequence alignment of $ALDH_{Tt}$ (PDB: 6FJX) and A) ALDH1A3 (PDB: 5FHZ) and B) succinate semialdehyde dehydrogenase (SS-ALDH) from E. coli (PDB: 3JZ4).

Table S1: Aldehyde substrates used for catalysis by $ALDH_{Tt}$ highlighting their associated specific activity.

**Chemical Structure**	**Substrate**	**Specific activity (U/mg)**	
		**25 °C**	**50 °C**
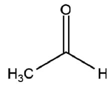	Acetaldehyde	0.006 ± 0.0003	0.098 ± 0.009
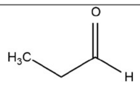	Propanal	0.01 ± 0.002	0.40 ± 0.01
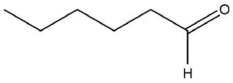	Hexanal	0.09 ± 0.002	1.08 ± 0.03
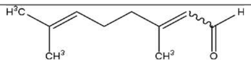	Citral	-	-
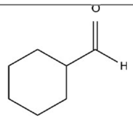	Cyclohexanecarbox-aldehyde	0.004 ± 0.007	0.11 ± 0.008
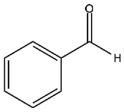	Benzaldehyde	0.03 ± 0.002	0.09 ± 0.008
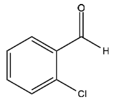	2-chlorobenzaldehyde	-	0.005 ± 0.004
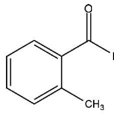	*o*-tolualdehyde	-	-
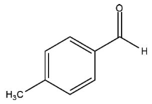	*p*-tolualdehyde	0.02 ± 0.003	0.23 ± 0.08
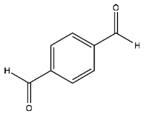	Terephthalaldehyde	0.09 ± 0.005	0.88 ± 0.05
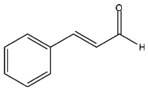	*trans*-Cinnamaldehyde	0.02 ± 0.01	0.12 ± 0.03
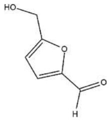	5-hydroxymethyl-2-furfural	-	-

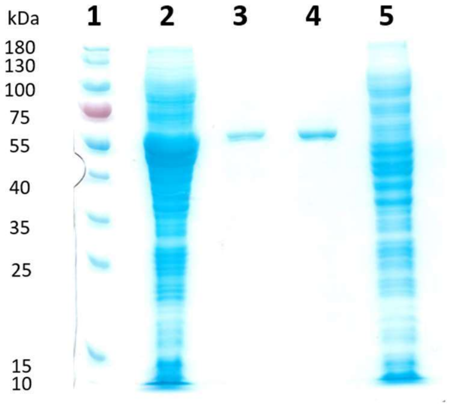

Figure S3: SDS-PAGE of $ALDH_{Tt}$ expression and purification fractions omitting the heat treatment step. Lane 1: PageRuler prestained protein ladder (ThermoFisher), lane 2: E. coli BL21(DE3) cell lysate expressing $ALDH_{Tt}$, lane 3: Ni affinity chromatography 200 mM imidazole elution, lane 4: purified $ALDH_{Tt}$,, lane 5: Ni affinity chromatography flow through.

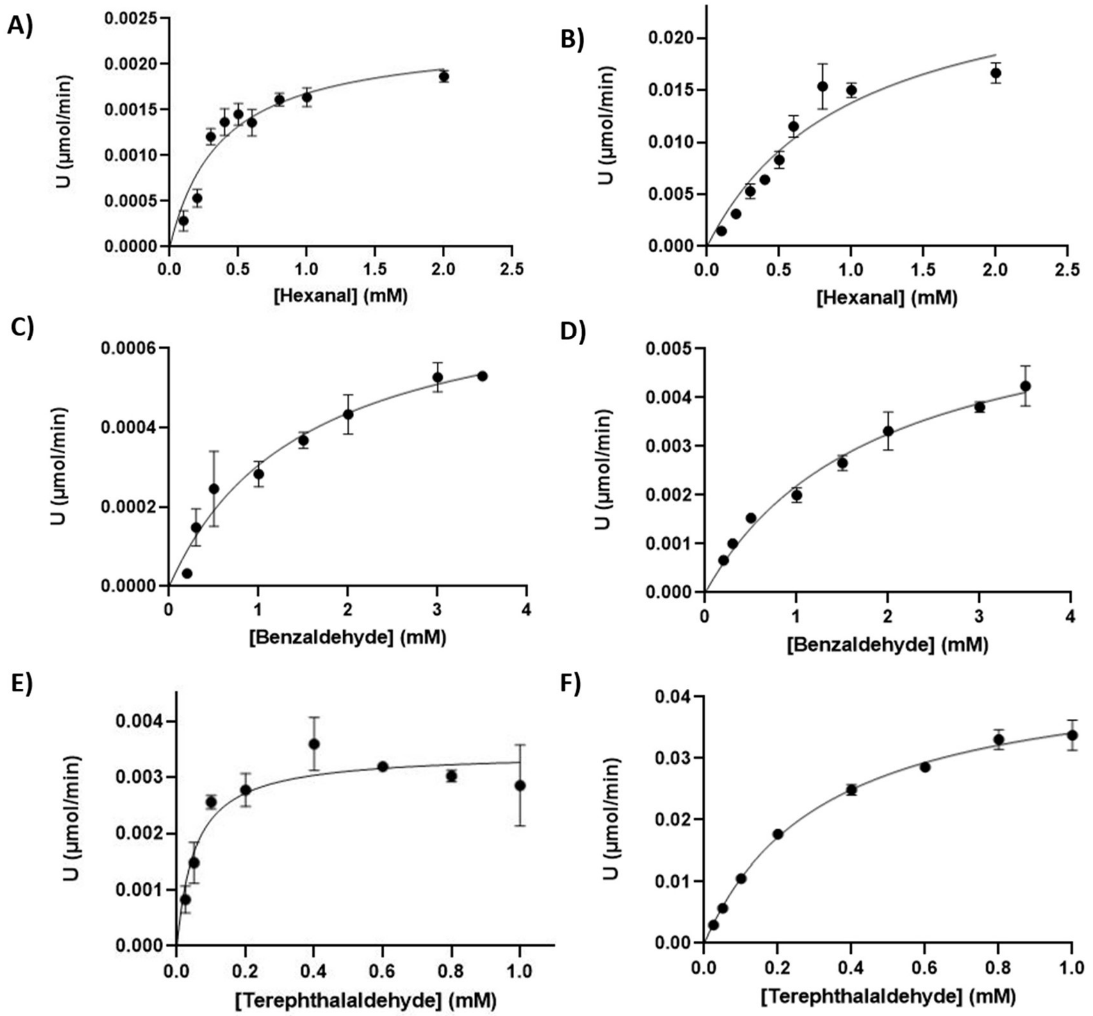

Figure S4: Michaelis-menten kinetic plots for $ALDH_{Tt}$ with different substrates, A) hexanal 25 °C, B) hexanal 50 °C C) benzaldehyde 25 °C, D) benzaldehyde 50 °C, E) terephthalaldehyde 25 °C, F) terephthalaldehyde 50 °C.

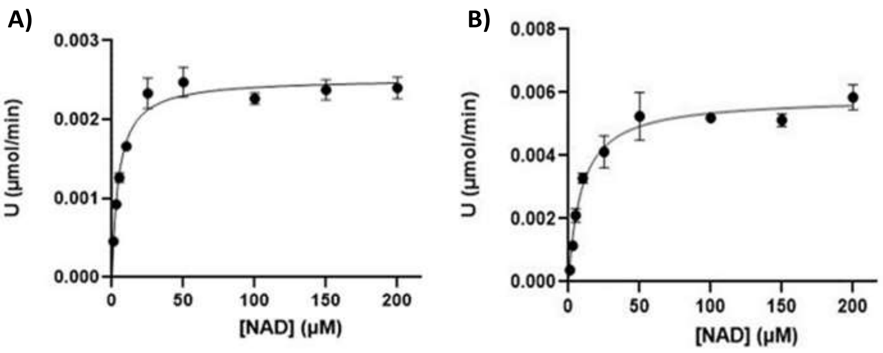

Figure S5: Michaelis-menten kinetics plot varying $[NAD^+]$ for $ALDH_{Tt}$ using hexanal at A) 25 °C and B) 50°C.

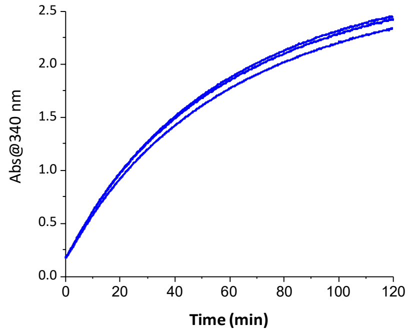

Figure S6: $ALDH_{Tt}$ reaction course over 2 h using hexanal with NADH production monitored at 340 nm.

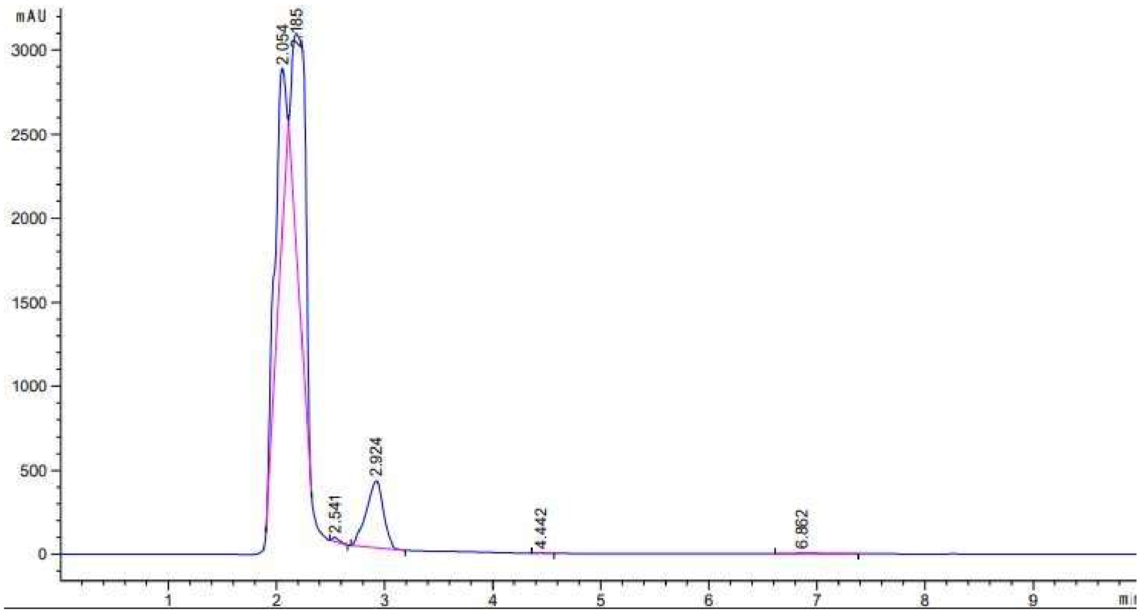

Figure S7: HPLC chromatogram for the detection of hexanoic acid from 2 h $ALDH_{Tt}$ assay with a retention time of 6.86 min.

Table S2: Esterase substrates catalysed by $ALDH_{Tt}$ highlighting their associated specific activity.

Chemical Structure	Substrate	Specific Activity (U/mg)
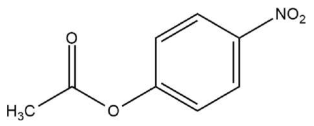	*p*-nitrophenyl acetate	0.033 ± 0.006
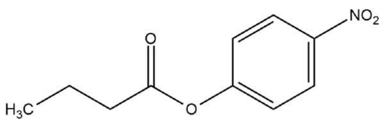	*p*-nitrophenyl butyrate	0.013 ± 0.0002

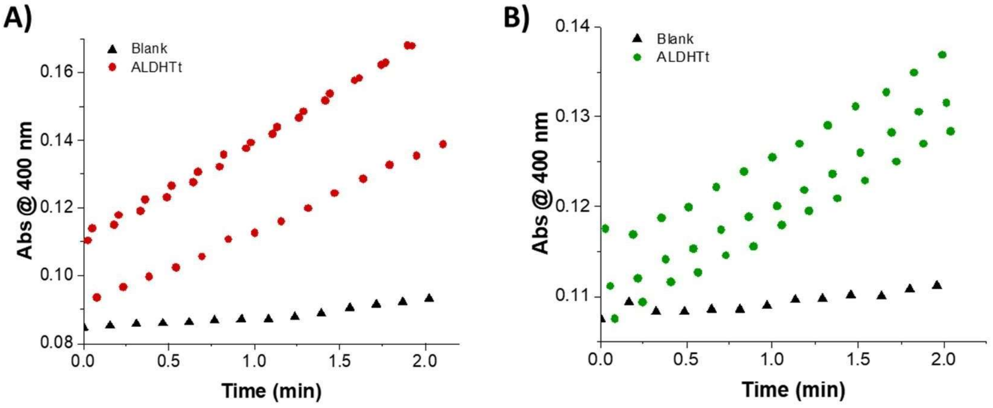

Figure S8: Absorbance vs. time for esterase activity monitored at 400 nm at 25°C using A) PNP-acetate and B) PNP-butyrate.

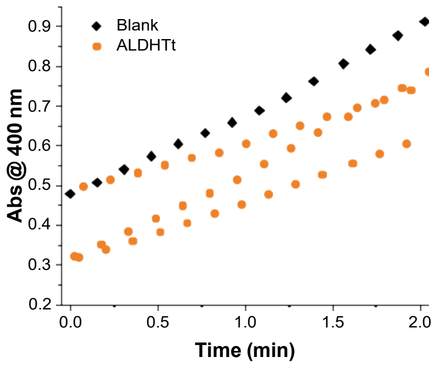

Figure S9: Absorbance vs. time monitored at 400 nm for esterase activity at 50°C using PNP-acetate.
